# Effects of Smoking on the Prevalence of Allergic Disorders in Russian Adolescents: A Retrospective Cross-sectional Study

**DOI:** 10.7759/cureus.3912

**Published:** 2019-01-18

**Authors:** Julia Levina, Valeriy Zvonarev, Leyla Namazova-Baranova

**Affiliations:** 1 Allergy and Immunology, Central Clinical Hospital of Russian Academy of Sciences, Moscow, RUS; 2 Psychiatry, Columbia Southern University, Orange Beach, USA; 3 Pediatrics, Central Clinical Hospital of Russian Academy of Sciences, Moscow, RUS

**Keywords:** atopic disorders, disease prevention, allergy, asthma, smoking, maternal smoking, prevalence, association

## Abstract

Introduction

Smoking is one of the leading causes of death. Smoking also decreases lung efficiency and impairs lung function in children of all ages. Second-hand tobacco smoking increases both the frequency and severity of childhood asthma. However, the role of active tobacco smoking in the manifestation of asthma remains inconclusive. The aim of this article was to describe the current understanding of the prevalence and adverse effects of cigarette smoking and to determine whether there is an association between smoking and the manifestation and progression of asthma among adolescents and high school students in Russia.

Objectives

This study aimed to determine the true prevalence of bronchial asthma (BA), allergic rhinitis (AR), and atopic dermatitis (AD) in the population of adolescents in the Russian Federation. In the GA^2^LEN (Global Allergy and Asthma European Network) study, we analyzed participant responses to confirm a connection between smoking and asthma exacerbation or development. This study examined the association between parental smoking and childhood atopic disorders. We sought to determine the effects of exposure to environmental tobacco smoke and childhood cigarette smoking on asthma symptoms among high school and college students in Moscow, Russia. We analyzed the reactions to the present tobacco control policy and evaluated the significance of the success of the policy. A number of methods were used, including questionnaires, surveys, and statistical analysis software.

Results

Our research indicates a moderate decrease in smoking prevalence among Russian teenagers. From the data and results of our study, 25.3% of the respondents said that they have ever smoked for as long as a year. Among the participants who had ever smoked, most of them started smoking at the age of 16 years while the state-reported average age for a new smoker is 11.4. Fifty percent of the respondents who had shortness of breath had been woken by an attack once a week in the last three months. Only 21.1% of the people with asthma were taking medicines for it. According to our calculations, there is no significant association between smoking status and the prevalence of asthma, hay fever, and sinusitis. More respondents (34.2%) had fathers who regularly smoked during their childhood than mothers who regularly smoked during their childhood or before the children were born (7.9%). There is a statistically significant association between a mother smoking during childhood or before the birth of a child and that child being hospitalized before the age of two years for lung disease.

Conclusion

The fact that the smoking rates among study participants are relatively low is quite encouraging and anti-smoking campaigns should be intensified to drive the rates lower. The data analyzed do not provide sufficient evidence to show that there is any causative association between smoking status and the development of asthma, hay fever, or sinusitis. Since there is no clear association between smoking status and the development of asthma, hay fever, or allergic sinusitis, it is imperative to say that smoking cessation will have very little effect on allergy prevalence as per the data analyzed. From the conducted analysis, it is evident that there is a clear link between second-hand smoke and asthma in children. According to our results, children whose mothers smoked are especially vulnerable to asthma and other respiratory disorders.

## Introduction

Smoking is a major cause of preventable deaths worldwide. In fact, many adults who smoke developed the habit when they were young. Unfortunately, Russia faces a heavy burden of tobacco consumption that has affected not only adults but also adolescents and high school students. Exposure to second-hand smoking increases the risk of developing asthma in childhood [1]. However, the role of active tobacco smoking in the manifestation of asthma remains inconclusive.

In a study of several schools in Russia, it was found that students who smoke or who are exposed to people who smoke are likely to suffer from asthma. Despite the fact that the sale and use of cigarettes or any nicotine-containing product that resembles cigarettes to and among individuals younger than 18 years old are prohibited in Russia [2], tobacco is still available and adolescents are likely to smoke.

According to the Centers for Disease Control and Prevention (CDC), environmental tobacco smoke exposure increases the risk of serious respiratory problems in children, such as a greater number and severity of asthma attacks and lower respiratory tract infections, and increases the risk of middle ear infections [3].

However, other studies, such as the 2004 Surgeon General's report, argue that there is no proof that smoking is the causative agent of asthma. The author highlights the inadequate proof in previous research on adults, which includes the difficulty in separating asthma from chronic obstructive pulmonary disease (COPD) in adult smokers and the lack of adequate evidence regarding asthma in relation to cigarette smokers. Furthermore, the author indicates a lack of proper research and data analysis approaches showing the link between smoking and other atopic disorders [3].

The purpose of this article was to describe the current understanding of the prevalence and adverse effects of cigarette smoking and to determine whether there is an association between smoking and the manifestation and progression of asthma among adolescents and high school students in Russia. We described the current understanding of the prevalence and adverse effects of cigarette smoking and second-hand smoke (SHS) in asthmatics in terms of patient outcomes and response to inhaled corticosteroids.

Understanding the nature and role of smoking in the development of atopic disorders and being able to use these data appropriately are critical for the development of national smoking prevention programs. Unfortunately, the results of even very well-structured state smoking prevention programs were not impressive. The aim of this study was to analyze the reactions to the present tobacco control policy and evaluate the significance of the success of the policy. We hope to assess the effectiveness of state school-based smoking prevention programs through the assessment of this study data. This can lead to changes in health practices, cultural patterns, and environmental impact. The paper also discussed the financial implications of smoking and its impact on society. Similarly, government responsibilities regarding maternal smoking in pregnancy (MSP) and policy implications regarding smoking and its ethical concerns were also addressed in this research article.

The prevention of exposure to tobacco smoke during pregnancy has been the sole factor that has consistently been shown to reduce the asthma risk in various populations and study settings in Russia. There has been little or no success shown by other primary means used to prevent asthma. This article also analyzed the effects of parental smoking throughout infancy, childhood, and adolescence with regards to asthma manifestation and progression.

Information on smoking prevalence will enable the administration to obtain a true picture of what proportion of the population smokes and the distribution across different segments of the population. To analyze smoking prevalence, a quantitative research design was used, which provided the opportunity to engage the sample through surveys, polls, or even questionnaires. Once data were collected, statistical and mathematical analysis methods were employed while providing for a margin of error [4]. The sample used in this study is representative of the population and any results found can be used to draw inferences on the smoking prevalence rates in Russia. We hope our findings will serve as a model for other research in Russia.

## Materials and methods

Study description

The objective was to determine the true prevalence of bronchial asthma (BA), allergic rhinitis (AR), and atopic dermatitis (AD) in the population of adolescents in the Russian Federation. We sought to determine the effects of exposure to environmental tobacco smoke and childhood cigarette smoking on asthma symptoms among high school and college students in Moscow, Russia. This study examines the association between parental smoking (during the pregnancy, in the postnatal period, and later) and childhood atopic disorders such as bronchial asthma, hay fever, and so on.

Stage I

A continuous sample of adolescents aged 15-18 years was created from our own databases according to the GA^2^LEN (Global Allergy and Asthma European Network) protocol in two research centers (Moscow and Tomsk, high level of urbanization). The external validity of the study is high.

• 12,803 adolescents (5000 in Moscow, 7803 in Tomsk) received an invitation to participate in research by mail (letters with a questionnaire).

• 2590 adolescents (1480 in Moscow, 1110 in Tomsk) answered and were included in statistical processing.

The cumulative morbidity of BA and AR was assessed in the case of positive answers to the questions: “Wheezing/whistling in your chest at any time in the last 12 months? Have you suffered from an allergy involving nasal problems (rhinitis, irritation, sneezing) in the last 12 months?”

The cumulative morbidity of AD was assessed in case of a positive answer to the question, “Did you ever have eczema or any kind of skin allergy?”

The prevalence of clinically diagnosed BA and AR was assessed when responders answered “yes” to the questions: “Have you ever had asthma? Was this confirmed by a doctor?” Have you ever had chronic rhinitis? Was this confirmed by a doctor?”

The prevalence of clinically diagnosed AD was assessed when responders answered “yes” to the question: “Did you ever have an itchy rash that was coming and going for at least six months?”

The cumulative morbidity of BA was marked at 19.9 % of all respondents included in the research. There were significantly more girls than boys with cumulative morbidity of BA registered in all populations of adolescents (61.2%, at р=0,039) and among children from Tomsk (68.2%, at р=0,003). The analysis of the prevalence of clinically diagnosed BA has shown that only 7.2% of teenagers had a medically verified diagnosis (2.7 times less in comparison with the cumulative morbidity of asthma) [2].

Stage II

A complex selective examination of 303 adolescents from Moscow (219 females and 84 males) was conducted.

The examination included:

- Spirometry, reversibility test (MasterScreen, Viasys Healthcare GmbH, Germany)

- Skin prick test (SPT) with 12 inhalant allergens (ALK-Scherax, Germany)

- Total immunoglobulin E (IgE) and specific IgE (sIgE) (ImmunoCap, Thermo Fisher Scientific, Uppsala, Sweden)

During the complex examination of the selected number of adolescents at the second stage of the study, the diagnosis of asthma was verified in 5.7% of children from the group with asthma-like symptoms and in 4.9% of children without asthma symptoms according to answers in the questionnaires (the average index was 5.1% of adolescents in the group) [5].

According to the official data, asthma morbidity was 2836.25 per 100,000. Thus, the true prevalence of asthma appears to be four times less than the prevalence of symptoms registered by patients, 1.4 times less than the “diagnosed illness” according to the answers of the patients, and two times above the official statistical data.

Stage III

In the GA^2^LEN study, we analyzed participant responses to confirm a connection between smoking and asthma exacerbation or development. Also, we searched the English biomedical literature via PubMed, Embase, and Scopus using the terms "smoking and asthma," "parental smoke and childhood asthma exacerbation," and "second-hand tobacco smoke and asthma." Those who participated in this research included high school students (13-17 years old), young adults (18-23 years old), their parents, primary investigators, and various research assistants. A number of methods were used, including questionnaires, surveys, and statistical analysis software.

Stage III of our research was conducted to ascertain the nationwide spread of asthma and its risk factors. The participants in this research were patients of the Partial Hospitalization Program at National Medical Research Centre for Children's Health, Moscow, Russia. The standard GA^2^LEN questionnaire was the main tool used in data collection, with additional standardized questions aimed at collecting information on symptoms and risk factors related to asthma. The respondents to these questionnaires were children who completed them during their regular check-up visits to the clinic. Since the subsequent questions depended on the previous question of the questionnaire, the questionnaires were subjected to scrutiny to discern the missing data as well as the correct skip patterns. For example, participants who stated that they do not smoke were expected to skip the following question that required them to quote the number of cigarettes they smoked in a day. In order to effectively conduct a statistical analysis, the researchers employed the Statistical Package for the Social Sciences (SPSS) v22.0 software (IBM Corp., Armonk, NY, US) for the interactive statistical analysis in our research.

Our team of researchers:

1. Conducted an analysis of smoking prevalence among study participants

2. Researched associations between smoking status and the prevalence of asthma, hay fever, or sinusitis

After the establishment of smoking patterns and the relation to the above health conditions, we also showed an interest in the examination of initial attempts by our participants to stop their smoking habits. Finally, we assessed the parental smoking habits and related this to the smoking habits of the children as well as to atopic disorders among the children. This was in an effort to establish whether some of the parental behaviors could be inherited by their offspring.

Children’s histories of cigarette smoking and exposure to environmental tobacco smoke (ETS) were estimated from their questionnaire responses as well as from their parents’ responses to a separate questionnaire. This was to confirm whether children exposed to a smoking environment can also become smokers. To achieve this, the children were asked if they normally smoked and those who gave affirmative answers were requested to mention the number of cigarettes they smoked per day. As this was not sufficient, the researchers were compelled to ask the children how many of their family members smoked and the number of days that they normally inhaled smoke in a month from those people who normally smoked. Through this investigation, it was apparent that there were no clear results on how many members of the family smoked, but we were able to collect data regarding a child’s exposure to maternal smoke before birth.

In order to come up with further findings, the investigators first asked the children if the physician had ever declared them asthmatic. They were also assigned pamphlets with a description of wheezing and allergies for them to read and were then asked to explain whether they had ever experienced wheezing in the previous 12 months or experienced any other atopic symptoms. To avoid misleading information, mixed sources of tobacco smoke were checked, as well as the link between asthma and the children’s exposure to smoking environments. This was considered in smoking children due to the influence of ETS. Nevertheless, all information on missing data and outcome variables were included in the final report of this research. This made it possible for us to have a final report, with data from the original 303 participants who were selected for the research.

In the end, we used the X2 test in the analysis of the data collected and to establish the relationships that existed between asthma and cigarette smoking.

In hypothesis testing, a null hypothesis (H0) and an alternative hypothesis (Ha) are contrasted. H0 states an argument that researchers try to disprove, reject, or nullify. H0 establishes no difference or no association between the dependent and the independent variables, whereas Ha establishes a difference or an association between the dependent and the independent variables. Ha may be one-sided or two-sided:

• With a two-sided hypothesis, the direction is not important; a two-sided Ha claims that a parameter is not equal to the value proposed by H0.

• With a one-sided hypothesis, the direction is important; a one-sided Ha claims that a population parameter is either larger than the value proposed by H0 or smaller than that value.

H0 often refers to the opposite of what researchers really believe is the cause of a phenomenon, whereas Ha is what researchers really think is the cause of a phenomenon. If the difference or association is found to be statistically significant, H0 can be rejected.

The p-value is used to strengthen the results of the study and represents the probability of observing a given result due to chance alone, assuming that the null hypothesis is true. The commonly accepted upper limit (cut-off point) of the p-value for a result to be considered statistically significant is 0.05; this means that we will reject the null hypothesis only if its probability of being true is less than 5%. A chi-square independence test evaluates if two categorical values are associated in the study population. This test can be employed for a relatively large sample size. Through the analysis of the relationship between smoke exposure and asthma symptoms, tobacco exposure was forecast by indicator variables. The total number of cigarettes smoked per day, amount of second-hand exposure per month, number of family smokers, and number smoked prior to cessation was also provided. From this study, it can be concluded that there is a possibility that smokers will develop asthmatic conditions. Second, children exposed to smoking environments have the possibility of becoming smokers.

## Results

Analysis of past medical history of bronchial asthma and other atopic disorders in study participants

From the results below, among the 296 respondents who validly responded to this question, only 5.1% had ever had asthma in their lifetime, with the majority (94.9%) indicating that they had never had asthma. The results in Table 1 indicate that among the 289 respondents who validly responded to this question, only 10.4% of them indicated that they had nasal allergies, including hay fever. The majority (89.6%) of the respondents indicated that they did not have any nasal allergies, including hay fever. From Table 1, among the 292 respondents who validly responded to this question, only 3.4% had ever been told by a doctor that they had chronic sinusitis or nasal polyps. The majority (96.6%) of the respondents had never been told that they have chronic sinusitis or nasal polyps. From Table 1, among the 282 individuals who validly responded to this question, a mere 3.9% had ever been hospitalized before the age of two years for lung disease. The majority (96.1%) had never been hospitalized before the age of two years for lung disease.

**Table 1:** Analysis of past medical history of bronchial asthma and other atopic disorders in study participants

Have you ever had asthma?
	Frequency	Percent	Valid Percent	Cumulative Percent
Valid	No	281	92.7	94.9	89.6
Yes	15	5,0	5.1	100.0
Total	296	97.7	100.0	
Missing	System	7	2.3		
Total	303	100.0		

Have any nasal allergies, including hay fever?
	Frequency	Percent	Valid Percent	Cumulative Percent
Valid	No	259	85.5	89.6	89.6
Yes	30	9.9	10.4	100.0
Total	289	95.4	100.0	
Missing	System	14	4.6		
Total	303	100.0		

Doctor ever told you have chronic sinusitis or nasal polyps?
	Frequency	Percent	Valid Percent	Cumulative Percent
Valid	No	282	93.l	96.6	96.6
Yes	10	3.3	3.4	100.0
Total	292	96.4	100.0	
Missing	System	11	3.6		
Total	303	100.0		

Hospitalized before the age of two years for lung disease?	
	Frequency	Percent	Valid Percent	Cumulative Percent
Valid	No	271	89.4	96.1	96.1
Yes.	11	3.6	3.9	100.0
Total	282	93.l	100.0	
Missing	System	21	6.9		
Total	303	100.0		

Prevalence of allergic diseases in the study population

Among the respondents, the results in Table 2 show that the majority (82.8%) had not experienced any wheezing/whistling in their chest at any time in the last 12 months. Among those who had experienced wheezing in the past 12 months, only 32.7% had been breathless when the wheezing sound was present. From Table 2, only 44.6% had wheezing or whistling when they did not have a cold. From Table 2, only 4% of the respondents had been woken up by an attack of shortness of breath in the last 12 months. Only 42.9% of the respondents had been woken up by an attack of shortness of breath in the last three months. Fifty percent of the respondents who had shortness of breath had been woken by an attack once a week in the last three months.

**Table 2:** Prevalence of allergic diseases in the study population

Wheezing/whistling in your chest at any time in the last 12 months?
	Frequency	Percent	Valid Percent	Cumulative Percent
Valid	No	250	82.5	82.8	82.8
Yes	52	17.2	17.2	100.0
Total	302	99.7	100.0	
Missing	System	1	.3		
Total	303	100.0		

Been at all breathless when the wheezing noise was present?
	Frequency	Percent	Valid Percent	Cumulative Percent
Valid	No	35	11.6	67.3	67.3
Yes	17	5.6	32.7	100.0
Total	52	17.2	100.0	
Missing	System	251	82.8		
Total	303	100.0		

Had this wheezing or whistling when you did not have a cold?
	Frequency	Percent	Valid Percent	Cumulative Percent
Valid	No	31	10.2	55.4	55.4
Yes	25	8.3	44.6	100.0
Total	56	18.5	100.0	
Missing	System	247	81.5		
Total	303	100.0		

Woken by an attack of shortness of breath in the last 12 months?
	Frequency	Percent	Valid Percent	Cumulative Percent
Valid	No	287	94.7	96.0	96.0
Yes	12	4.0	4.0	100.0
Total	299	98.7	100.0	
Missing	System	4	1.3		
Total	303	100.0		

Woken by an attack of shortness of breath in the last 3 months?
	Frequency	Percent	Valid Percent	Cumulative Percent
Valid	No	8	2.6	57.1	57.1
Yes	6	2.0	42.9	100.0
Total	14	4.6	100.0	
Missing	System	289	95.4		
Total	303	100.0		

Statistics
Times a week on average woken by shortness of breath in the last 3 months?
N	Valid	1
Missing	104
Mean	1.00
Mode	1

Woken by an attack of coughing at any time in the last 12 months?
	Frequency	Percent	Valid Percent	Cumulative Percent
Valid	No	251	82.8	84.8	84.8
Yes	45	14.9	15.2	100.0
Total	296	97.7	100.0	
Missing	System	7	2.3		
Total	303	100.0		

Have you ever had asthma?
	Frequency	Percent	Valid Percent	Cumulative Percent
Valid	No	281	92.7	94.9	94.9
Yes	15	5.0	5.1	100.0
Total	296	97.7	100.0	
Missing	System	7	2.3		
Total	303	100.0		

Was this confirmed by a doctor?
	Frequency	Percent	Valid Percent	Cumulative Percent
Valid	No	2	.7	13.3	13.3
Yes	13	4.3	86.7	100.0
Total	15	5.0	100.0	
Missing	System	288	95.0		
Total	303	100.0		

Have you had an attack of asthma in the last 12 months?
	Frequency	Percent	Valid Percent	Cumulative Percent
Valid	No	11	3.6	73.3	73.3
Yes	4	1.3	26.7	100.0
Total	15	5.0	100.0	
Missing	System	288	95.0		
Total	303	100.0		

Statistics
	How old were you when you had your first attack of asthma? ## YEARS	How old were you when you had your most recent attack of asthma?## YEARS	How many attacks of asthma have you had in the last 12 months?#### ATTACKS	How many attacks of asthma have you had in the last 3 months?### ATTACKS	How many times have woken up because of asthma in last 3 months?	How often had trouble with breathing because of asthma last 3 months?
N	Valid	14	15	4	4	10	13
Missing	289	288	299	299	293	290
Mean	8.57	15.33	1.75	6.75	4.70	4.31
Mode	5^a^	16	0^a^	1	5	5
Std. Deviation	3.777	2.093	1.708	9.032	0.675	0.855
a. Multiple modes exist. The smallest value is shown

Are you taking any medicines for asthma?
	Frequency	Percent	Valid Percent	Cumulative Percent
Valid	No	15	5.0	78.9	78.9
Yes	4	1.3	21.1	100.0
Total	19	6.3	100.0	
Missing	System	284	93.7		
Total	303	100.0		

Statistics
	How old were when you first had sinusitis/nasal polyps?# Years	How old when first diagnosed as having chronic sinusitis/nasal polyps? ## YEARS	Age when first started to use antibiotics for nasal/sinus problems?# Years	On average how many months each year have you taken them?Months	How many of the last 5 years taken antibiotics for nasal/sinus? #Years	On average how many months of each of these years taken them?Months
N	Valid	9	7	4	1	3	2
Missing	294	296	299	302	300	301
Mean	12.78	13.57	7.50	1.00	1.00	1.00
Mode	14	14^a^	1^a^	1	0^a^	1
Std. Deviation	3.383	2.225	6.351		1.000	.000
a. Multiple modes exist. The smallest value is shown

Among the respondents who had suffered a shortness-of-breath attack in the last three months, they had, on average, been woken up by the shortness of breath once in the last three months. Only 15.2% of the respondents had been woken up by a coughing attack in the last 12 months. Finally, from Table 2 above, only 5.1% of the respondents have ever had asthma. Among the respondents who had asthma, 86.7% had asthma confirmed by a doctor. It is clear that only 26.7% of people who have ever had asthma had suffered an asthma attack in the last 12 months.

From Table 2, the respondents had their first asthma attack at the age of 8.57 years on average, and the respondents were 15.33 years old on average when they had the most recent asthma attack. On average, the respondents had 1.75 asthma attacks over the past 12 months. In the past three months, the respondents had had an average of 6.75 asthma attacks and had woken up due to an asthma attack an average of 4.7 times. On average, respondents with asthma had trouble breathing 4.31 times due to asthma in the last three months. Only 21.1% of the people with asthma were taking medicine for it.

Among those respondents who had sinusitis/nasal polyps, they first experienced it at an average age of 12.78 years but were first diagnosed with chronic sinusitis/nasal polyps at the age of 13.57 years. These respondents started using antibiotics at an average age of 7.5 years and had taken them for an average of one month per year. For the past five years, the respondents have taken antibiotics for an average of one year with an average of one month every year.

Analysis of smoking prevalence among study participants

From the data and results in Table 3, 25.3% of the respondents said that they had ever smoked for as long as a year. Among the respondents who had ever smoked, most started smoking at the age of 16 years. However, the average age that the respondents started smoking was 15.35 years. It shows that of the respondents who smoked, 68% still smoked as of one month ago.

**Table 3:** Analysis of smoking prevalence among study participants

Have you ever smoked for as long as a year?
	Frequency	Percent	Valid Percent	Cumulative Percent
Valid	No	221	72.9	74.7	74.7
Yes	75	24.8	25.3	100.0
Total	296	97.7	100.0	
Missing	System	7	2.3		
Total	303	100.0		

How old were you when you started smoking? #Years
N	Valid	74
Missing	229
Mean	15.35
Mode	16
Std. Deviation	1.708

Do you now smoke, as of one month ago?
	Frequency	Percent	Valid Percent	Cumulative Percent
Valid	No	24	7.9	32.0	32.0
Yes	51	16.8	68.0	100.0
Total	75	24.8	100.0	
Missing	System	228	75.2		
Total	303	100.0		

Statistics
	number of cigarettes per day	number of cigarillos per day	number of cigars a week	pipe tobacco in ounces/week ##ounces	pipe tobacco in grams/week ### grams
N	Valid	52	17	17	15	13
Missing	251	286	286	288	290
Mean	9.67	1.35	0.18	7.13	0.23
Mode	10	0	0	0	0
Std. Deviation	6.170	4.834	0.529	23.339	0.832

Have you stopped or cut down smoking?
	Frequency	Percent	Valid Percent	Cumulative Percent
Valid	No	31	10.2	44.9	44.9
Yes	38	12.5	55.1	100.0
Total	69	22.8	100.0	
Missing	System	234	77.2		
Total	303	100.0		

Statistics
How old were you when you stopped or cut down smoking? ## YEARS
N	Valid	37
Missing	266
Mean	17.51
Mode	18
Std. Deviation	1.070

Statistics
	number of cigarettes per day	number of cigarillos per day	number of cigars a week	pipe tobacco in ounces/week	pipe tobacco in grams/week ### grams
N	Valid	36	14	14	14	12
Missing	267	289	289	289	291
Mean	11.81	0.00	0.14	0.00	0.00
Mode	10	0	0	0	0
Std. Deviation	6.337	0.000	0.535	0.000	0.000

Regularly exposed to tobacco smoke in the last 12 months?
	Frequency	Percent	Valid Percent	Cumulative Percent
Valid	No	212	70.0	73.1	73.1
Yes	78	25.7	26.9	100.0
Total	290	95.7	100.0	
Missing	System	13	4.3		
Total	303	100.0		

Statistics
How many people in your household smoke regularly?
N	Valid	78
Missing	225
Mean	1.26
Mode	1
Std. Deviation	1.362

Do people smoke regularly in the room where you work?
	Frequency	Percent	Valid Percent	Cumulative Percent
Valid	No	68	22.4	87.2	87.2
Yes	10	3.3	12.8	100.0
Total	78	25.7	100.0	
Missing	System	225	74.3		
Total	303	100.0		

Statistics
	At home (Hours)	At workplace	In bars, restaurants, cinemas or similar social settings	Elsewhere
N	Valid	33	30	32	36
Missing	270	273	271	267
Mean	2.33	1.97	1.69	1.72
Mode	0	0	1	0^a^
Std. Deviation	2.901	2.428	1.533	1.892
a. Multiple modes exist. The smallest value is shown

For the respondents that were still smoking as of one month ago, they smoked 9.67 cigarettes on average per day and most smoked 10 cigarettes per day. The average number of cigarettes smoked per day was 1.35. On average, the respondents smoked 0.18 cigars a week, 7.13 ounces of pipe tobacco per week, and 0.23 grams of pipe tobacco per week. It is evident that among those who had been engaged in smoking, 55.1% had already stopped or cut down on smoking. From Table 3 above, as much as the majority of respondents had either stopped or cut down smoking at the age of 18 years, the average age at which the respondents stopped or cut down their smoking was 17.51 years.

Among those who had stopped or cut down their smoking, they smoked an average of 11.81 cigarettes a day, zero cigarillos per day, and 0.14 cigars a week, with zero ounces of pipe tobacco per week and zero grams of piped tobacco.

Among those included in Table 3 above, 83.9% did or do inhale smoke. The majority of respondents (73.1%) have not been regularly exposed to tobacco in the last 12 months.

For those people who have been exposed to smoke in the past 12 months, they had, on average, 1.26 family members in their household who smoked regularly. From the results in Table 3, it is clear that the majority of respondents (87.2%) did not encounter people smoking regularly in the room in which they worked. Among the respondents who were exposed to other people’s smoke, they were, on average, exposed to other people’s tobacco smoke for 2.33 hours at home, 1.97 hours at work, 1.69 in bars, restaurants, cinemas, or social settings, and 1.72 hours elsewhere.

Analysis of smoking cessation attempts in this population

Only 55.1% of the smokers had either stopped or cut down. Information from Table 4 indicates that the average age at which the respondents had stopped or cut down on smoking was 17.51 years.

**Table 4:** Analysis of smoking cessation attempts in this population

Have you stopped or cut down smoking?
	Frequency	Percent	Valid Percent	Cumulative Percent
Valid	No	31	10.2	44.9	44.9
Yes	38	12.5	55.1	100.0
Total	69	22.8	100.0	
Missing	System	234	77.2		
Total	303	100.0		

Associations between smoking status and the prevalence of asthma, hay fever, or sinusitis

From Table 5, the Pearson chi-square p-value is 0.935, which is greater than the level of significance of 0.05. Therefore, there is no significant association between smoking status and the prevalence of asthma. A chi-square test was conducted, and the Pearson chi-square p-value was 0.465; this is greater than the level of significance of 0.05, which shows that there is no significant association between smoking status and the prevalence of hay fever. The results in Table 5 show that the Pearson chi-square p-value is 0.688. This p-value is greater than the level of significance of 0.05. Therefore, this indicates that there is no statistically significant association between smoking status and sinusitis.

**Table 5:** Associations between smoking status and the prevalence of asthma, hay fever, or sinusitis

Chi-Square Tests (asthma)
	Value	df	Asymp. Sig. (2-sided)	Exact Sig. (2-sided)	Exact Sig. (1-sided)
Pearson Chi-Square	0.007^a^	1	0.935		
Continuity Correction^b^	0.000	1	1.000		
Likelihood Ratio	0.007	1	0.935		
Fisher's Exact Test				1.000	0.570
Linear-by-Linear Association	0.007	1	0.935		
N of Valid Cases	291				
a. 1 cells (25.0%) have expected count less than 5. The minimum expected count is 3.87.
b. Computed only for a 2x2 table

Chi-Square Tests (hay fever)
	Value	df	Asymp. Sig. (2-sided)	Exact Sig. (2-sided)	Exact Sig. (1-sided)
Pearson Chi-Square	0.533^a^	1	0.465		
Continuity Correction^b^	0.255	1	0.614		
Likelihood Ratio	0.513	1	0.474		
Fisher's Exact Test				.501	.299
Linear-by-Linear Association	0.531	1	0.466		
N of Valid Cases	283				
a. 0 cells (0.0%) have expected count less than 5. The minimum expected count is 7.38.
b. Computed only for a 2x2 table

Chi-Square Tests (sinusitis)
	Value	df	Asymp. Sig. (2-sided)	Exact Sig. (2-sided)	Exact Sig. (1-sided)
Pearson Chi-Square	0.161^a^	1	0.688		
Continuity Correction^b^	0.001	1	0.974		
Likelihood Ratio	0.170	1	0.680		
Fisher's Exact Test				1.000	0.511
Linear-by-Linear Association	0.161	1	0.688		
N of Valid Cases	287				
a. 1 cells (25.0%) have an expected count of less than 5. The minimum expected count is 2.54.
b. Computed only for a 2x2 table

Parental smoking and childhood asthma 

Generally, the smoking prevalence rates among women have always been significantly lower as compared to men worldwide. However, in later years, it has been noted through research that smoking among women in Western countries is higher than in women in Asia. Juch-Choi et al. indicated that before the Civil War and World War I, men mostly smoked while women tend to be engaged in pipe smoking. However, the number of women smokers has increased over the years across the world [6].

From Table 6, among the 295 respondents who validly answered this question, only 34.2% of respondents said that their father had smoked regularly during their childhood. The majority of respondents did not have fathers who smoked regularly during their childhood. Among the 291 individuals who validly responded to this question, 7.9% had mothers who had ever smoked regularly during/before they were born. The majority (87.6%) of the respondents had mothers who had never smoked regularly during their childhood or before they were born. More respondents (34.2%) had fathers who smoked regularly during their childhood than mothers who regularly smoked during their childhood or before they were born (7.9%).

**Table 6:** Parental smoking and childhood asthma

Did your father ever smoke regularly during your childhood?
	Frequency	Percent	Valid Percent	Cumulative Percent
Valid	No	158	52.l	53.6	53.6
Yes	101	33.3	34.2	87.8
Don't Know	36	11.9	12.2	100.0
Total	295	97.4	100.0	
Missing	System	8	2.6		
Total	303	100.0		

Mother ever smoke regularly during childhood/before you were born?
	Frequency	Percent	Valid Percent	Cumulative Percent
Valid	No	255	84.2	87.6	87.6
Yes	23	7.6	7.9	95.5
Don't Know	13	4.3	4,5	100.0
Total	291	96.0	100.0	
Missing	System	12	4.0		
Total	303	100.0		

Association between father regularly smoking during a child’s childhood and the child having asthma

From the chi-square results in Table 7, the Pearson chi-squared p-value of 0.532 is greater than the level of significance of 0.05. Therefore, from the results, the null hypothesis is rejected, and the conclusion is that there is no significant association between childhood asthma and having a father who had ever smoking regularly during their childhood.

**Table 7:** Association between father regularly smoking during a child’s childhood and the child having asthma

Chi-Square Tests
	Value	df	Asymp. Sig. (2-sided)
Pearson Chi-Square	1.261^a^	2	0.532
Likelihood Ratio	1.258	2	0.533
Linear-by-Linear Association	0.018	1	0.892
N of Valid Cases	288		
a. 1 cells (16.7%) have expected count less than 5. The minimum expected count is 1.77.

Association between mother regularly smoking during childhood or before a child’s birth and the child having asthma

From Table 8, the chi-square results indicate that the p-value (0.613) associated with the Pearson chi-square is greater than the level of significance of 0.05. Therefore, the null hypothesis is rejected and the conclusion that there is no significant association between a mother smoking during childhood or before their birth and the child having asthma.

**Table 8:** Association between mother regularly smoking during childhood or before the child’s birth and the child having asthma

Chi-Square Tests
Value	df	Asymp. Sig. (2-sided)	
0.977^a^	2	0.613	
0.841	2	0.657	
0.785	1	0.376	
284			
a. 2 cells (33.3%) have expected count less than 5. The minimum expected count is 0.63.

Parental smoking and prevalence of nasal allergies among children

Association Between Father Regularly Smoking During Childhood and the Prevalence of Nasal Allergies in the Child

From the results in Table 9, it shows that the p-value (0.193) associated with the Pearson chi-square value is greater than the level of significance of 0.05. Therefore, we reject the null hypothesis and conclude that there is no association between a father smoking regularly during childhood and the prevalence of nasal allergies in the child*.*

**Table 9:** Parental smoking and prevalence of nasal allergies among children

Chi-Square Tests
	Value	df	Asymp. Sig. (2-sided)
Pearson Chi-Square	3.294^a^	2	0.193
Likelihood Ratio	4.059	2	0.131
Linear-by-Linear Association	3.143	1	0.076
N of Valid Cases	282		
a. 1 cells (16.7%) have expected count less than 5. The minimum expected count is 3.72.

Association Between Mother Regularly Smoking During a Child’s Childhood and the Prevalence of Nasal Allergies in the Child

From the results in Table 10, the p-value (0.594) associated with the Pearson Chi-square value is greater than the level of significance of 0.05. Therefore, we reject the null hypothesis and conclude that there is no association between a mother smoking regularly during childhood or before birth and the prevalence of nasal allergies in the child.

**Table 10:** Association between mother regularly smoking during a child’s childhood and the prevalence of nasal allergies in the child

Chi-Square Tests
	Value	df	Asymp. Sig. (2-sided)
Pearson Chi-Square	1.041^a^	2	0.594
Likelihood Ratio	1.239	2	0.538
Linear-by-Linear Association	0.683	1	0.409
N of Valid Cases	278		
a. 2 cells (33.3%) have expected count less than 5. The minimum expected count is 1.40.

Parental smoking and prevalence of chronic sinusitis or nasal polyps among children

*Association Between Father Regularly Smoking During a Child's Childhood and the Child Having Chronic Sinusitis or Nasal Polyps* 

From Table 11 below, the p-value (0.647) associated with the Pearson chi-square value is greater than the level of significance of 0.05. Therefore, we reject the null hypothesis and conclude that there is no statistically significant association between a father smoking regularly during childhood and that child developing chronic sinusitis or nasal polyps.

**Table 11:** Association between a father regularly smoking during childhood and the child having chronic sinusitis or nasal polyps

Chi-Square Tests
	Value	df	Asymp. Sig. (2-sided)
Pearson Chi-Square	0.870^a^	2	0.647
Likelihood Ratio	0.830	2	0.660
Linear-by-Linear Association	0.867	1	0.352
N of Valid Cases	285		
a. 2 cells (33.3%) have an expected count less than 5. The minimum expected count is 1.26.

Association Between Mother Regularly Smoking During Childhood or Before Birth and the Child Having Chronic Sinusitis or Nasal Polyps

From Table 12, the p-value (0.739) associated with the Pearson chi-square value is greater than the level of significance of 0.05. Therefore, we reject the null hypothesis and conclude that there is no statistically significant association between a mother smoking regularly during a child’s childhood or before birth and that child developing chronic sinusitis or nasal polyps.

**Table 12:** Association between mother regularly smoking during childhood or before birth and the child having chronic sinusitis or nasal polyps

Chi-Square Tests
	Value	df	Asymp. Sig. (2-sided)
Pearson Chi-Square	0.604^a^	2	0.739
Likelihood Ratio	1.054	2	0.590
Linear-by-Linear Association	0.183	1	0.669
N of Valid Cases	281		
a. 2 cells (33.3%) have expected count less than 5. The minimum expected count is .046.

Parental smoking and a child being hospitalized before the age of two years for lung disease

Association Between Father Regularly Smoking During Childhood and the Child Being Hospitalized Before the Age of Two Years for Lung Disease

From Table 13, the results indicate the p-value (0.410) associated with the Pearson chi-square value. Therefore, the null hypothesis is rejected, and the conclusion is that there is no statistically significant association between a father smoking during childhood and that child being hospitalized before the age of two years for lung disease.

**Table 13:** Parental smoking and a child being hospitalized before the age of two years for lung disease

Chi-Square Tests
	Value	df	Asymp. Sig. (2-sided)
Pearson Chi-Square	1.785^a^	2	0.410
Likelihood Ratio	3.080	2	0.214
Linear-by-Linear Association	0.414	1	0.520
N of Valid Cases	281		
a. 2 cells (33.3%) have expected count less than 5. The minimum expected count is 1.33.

Association Between Mother Regularly Smoking During Childhood or Before the Child’s Birth and the Child Being Hospitalized Before the Age of Two Years for Lung Disease

More specifically, in the SPSS output, you will find under Asymp. Sig. (2-sided), a value of 0.001. If the “Asymp. Sig. (2-sided)” for the Pearson chi-square statistic is less than 0.05, there is a relationship between the variables based on the level of confidence we stated in the beginning. 

Table 14 results indicate that the p-value (0.001) associated with the Pearson chi-square value is less than the level of significance of 0.05. Therefore, the null hypothesis is rejected, and we conclude that there is a statistically significant association between a mother smoking during childhood or before the birth of a child and that child being hospitalized before the age of two years for lung disease.

**Table 14:** Association between mother regularly smoking during childhood or before the child’s birth and the child being hospitalized before the age of two years for lung disease

Chi-Square Tests
	Value	df	Asymp. Sig. (2-sided)
Pearson Chi-Square	13.942^a^	2	0.001
Likelihood Ratio	8.567	2	0.014
Linear-by-Linear Association	2.729	1	0.099
N of Valid Cases	278		
a. 2 cells (33.3%) have expected count less than 5. The minimum expected count is 0.40.

## Discussion

According to previous studies, there is a relationship between smoking and the development and progression of bronchial asthma. Smokers have a higher risk of poorly controlled asthma as compared to non-smokers. In our experience, smokers are often excluded from randomized clinical trials, limiting data about the effective management of asthma in the smoking population. Several studies suggest that adults and teenagers who smoke are more likely to develop asthma, and there is strong evidence to incriminate second-hand smoke as well. Children who are around people who smoke have a higher chance of developing asthma in early life.

Choi and Bernat, in their research study, suggested that there is a relatively increased association of smoking with childhood asthma [7]. According to the study that was conducted in Florida, smoking was highly associated with childhood asthma among most children. As revealed in the results of the study, it was discovered that the prevalence of asthma was relatively higher among adolescents exposed to smoking than in their counterparts who were not. Over the last 30 days of the research study, it was discovered that cigarette smoking was highly associated with susceptibility among the asthmatic participants [7].

Exposure to environmental tobacco smoking (ETS) is deemed to be a key agent in the development and progression of asthma in teenagers. ETS decreases lung efficiency, impairs lung function, and increases both the frequency and severity of childhood asthma. According to the CDC, ETS aggravates sinusitis and cystic fibrosis and increases the likelihood of bronchitis and pneumonia [8].

Even though the underlying pathophysiological association between smoking and asthma is not yet known, a number of plausible mechanisms have been developed to help explain the entire process. First, smoking activates the elevated inflammatory reactions as well as reduces the immune systems, thus making these patients vulnerable to any respiratory attacks [9]. Second, smoking is associated with mucus hypersecretion and significantly reduces mucociliary transport [10]. Finally, “smoking asthma” has been associated with the neutrophilic infiltration of the lung tissue. These critical studies, among others, have continued to provide possible reasons why there is a relatively high prevalence of asthma among children who are exposed to smoking [7].

Maternal smoking in pregnancy (MSP) is a well-known risk factor for prenatal mortality, premature birth, low birth-weight, and small fetuses [6]. Asthma frequencies in children and adolescents have been proven to be increased by maternal smoking in pregnancy [11].

Gupta et al. reiterate the association between smoking and the development and progression of asthma. Researchers proved that there is increased development of asthma among smokers. Their results showed that the symptoms of asthma were positively associated with smoking [12].

The management options of asthma among smokers include higher-dose inhaled corticosteroids, targeting the small airways with extra-fine inhaled corticosteroids (ICS) formulations, leukotriene receptor antagonists, and combination therapy. A recent, randomized, double-blind study of 95 people with a mild form of asthma suggested that beclomethasone 2000 µg daily may improve control. Notably, after 12 weeks of therapy, there were no significant differences in peak expiratory flow or exacerbation rates in smokers as compared with non-smokers. Other authors state that smoking enhances the rapid clearance of theophylline because of the increased induction of the P450 metabolizing enzymes. Smoking is associated with deteriorating lung function and resistance to corticosteroids [13].

Price et al. provided an analysis of the leukotriene receptor antagonist (LTRA, montelukast) effect for the treatment of bronchial asthma among active and passive smokers. The largest RCT of smokers with asthma (31 countries) randomized patients to montelukast 10 mg daily (n=347), fluticasone propionate 250 μg twice daily (n=336), or placebo (n=336). At six months: the fluticasone and montelukast groups had significantly improved asthma control vs. the placebo (44.97% vs. 39.05%, respectively, p=0.04). Patients with a history of ≤11 pack years showed greater improvement with fluticasone; those with ≥11 pack years showed greater improvement with montelukast. As a result of these clear pieces of evidence, it will be critical for parents, the federal government, and interested non-government bodies to take care to prevent the possible exposure of these children to smoking [13].

Nowadays, more and more young people are engaging in tobacco smoking. The smoking of tobacco has become common in Russia but the government is working to reduce the availability of smoking tobacco in the country. Adolescents and high school students who smoke are more likely to get asthma and other, related disorders [14]. Furthermore, adolescents who start smoking when they are young are likely to continue with the habit, even in adulthood, which will affect their health and the health of their children [15]. Also, the government has noticed that the rate of young death is high because of smoking and has, therefore, come up with a way to restrict smoking by developing laws against smoking.

Smoking among adolescents in Russia

Tobacco smoking tends to vary from one country to another, one city to another, and one community to another. From 1986 to 1991, there was an increase in the prevalence of daily smoking among students of the Moscow school aged between 11 and 16 years. Besides, there was an increase in the weekly smoking rate among girls. From 1983 to 1989, research was carried out on ways to prevent smoking among students of the Moscow school. The preventive measures that the researchers employed during the study revealed that these students were indeed abundantly affected by the habit [16].

The prevalence of smoking was higher among boys as compared to girls [17]. It was actually 26.1% among the boys as compared to 5.7% among the girls. The Russia Longitudinal Monitoring Survey's round 13 gave out these data after doing a survey in 2004. The composition of the sample included 815 adolescents, whereby 385 were girls and 430 were boys. Their ages ranged from 14 to 17 years and they helped by providing answers regarding their health habits. Both sexes admitted to having experienced the use of alcohol in adolescence and maternal smoking too [18]. Girls mainly started smoking due to harsh socioeconomic conditions while boys were influenced by unstable families, low self-esteem, and physical inactivity [14].

**Figure 1:**
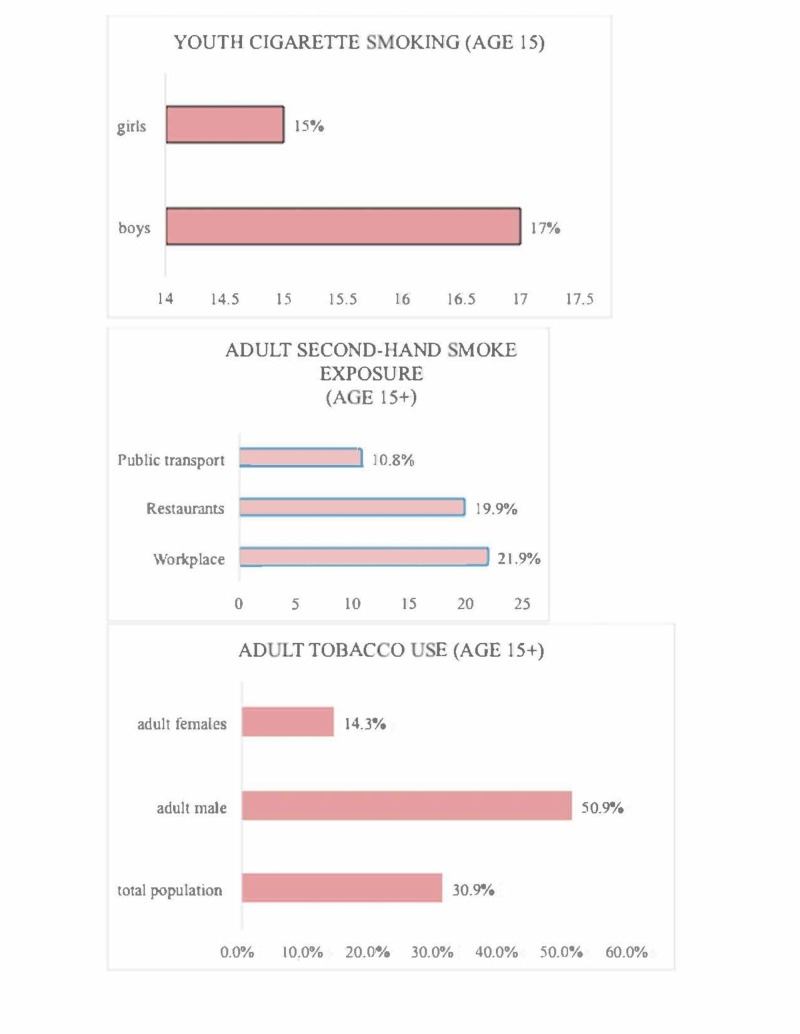
Smoking among adolescents in Russia

According to the Russian Federation Global Adult Tobacco Survey (GATS) (2017), 30.9% of Russians aged 15 and above use tobacco [19]. This is an indication that a large proportion of Russians are exposed to the direct or indirect consumption of tobacco. Similarly, nearly 17% of boys and 10% of girls aged 15 years smoke cigarettes in Russia. The results also indicate that 30% of the boys and 22% of the girls who used tobacco reported to have started smoking as early as 13 years or even younger. Also, 89% of youths aged between 13 and 15 are open to indirect tobacco smoke in community areas while 76% of young people are exposed to smoke at their homestead [20]. This means that even though someone might not be a smoker, he or she might be exposed to cigarette smoke in areas of residence or work. Figure 1 shows a simplified version of the data mentioned earlier.

To reach a valid conclusion, additional data was obtained from Health Behavior in School-Aged Children (HBSC), which is a World Health Organization (WHO) study that asks young people of age 11, 13, and 15 about their health and well-being in terms of their social life and health conditions in a self-survey report that occurs every four years [21]. Data were collected from different countries using a random sampling method for young adolescents of 11, 13, and 15 years, to ensure that the sample obtained is representative of the age range.

**Figure 2:**
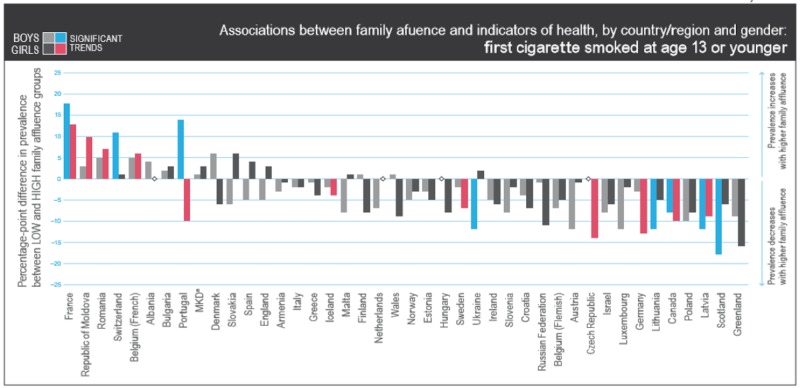
Association between family affluence and indicators of health, by country or regions and gender, for children who first smoked cigarettes at the age of 13 or younger Copyright © World Health Organization (WHO) Regional Office for Europe, 2016. All rights reserved. Images and other multimedia content used with permission.

Figure 2 shows the association between family affluence and indicators of health, by country or regions and gender, for children who first smoked cigarettes at the age of 13 or younger. In this bar chart, the proportion of Russian teenagers smoking cigarettes is low among families with high affluence as denoted by the bars below the 0% line. Prevalence is also low in countries such as Croatia and Italy, but the differences in Italy are small and there is no statistically significant linear trend. Although Greece and Scotland also have the same trend, it is statistically significant in boys in Scotland only.

**Figure 3:**
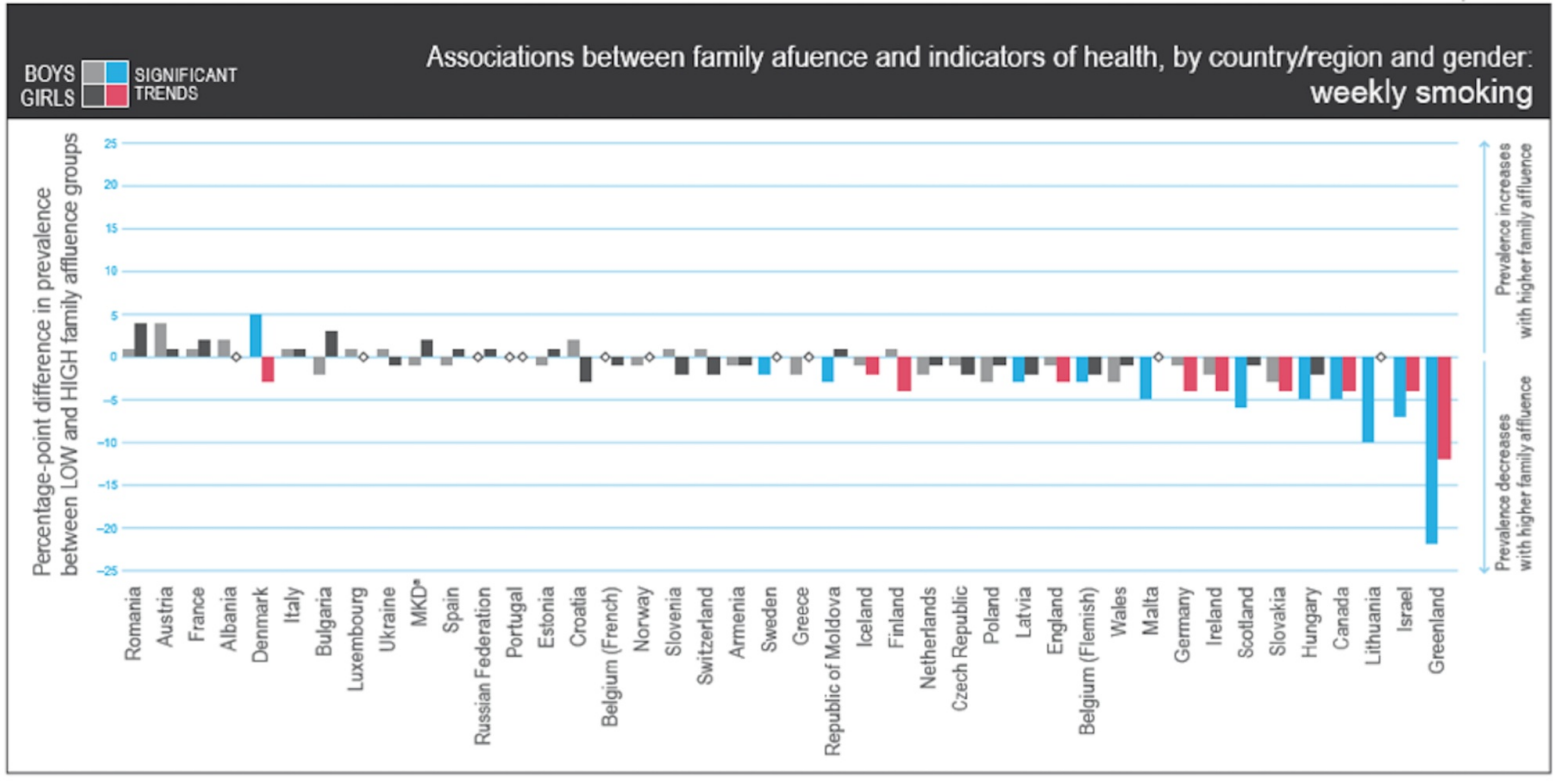
Associations between family affluence and indicators of health, by region or country and gender Copyright © World Health Organization (WHO) Regional Office for Europe, 2016. All rights reserved. Images and other multimedia content used with permission.

Figure 3 indicates associations between family affluence and indicators of health, by region or country and gender, for those adolescents who smoke weekly with categories ranging from never to every day. The bar chart in this section provides an overview of young children who prefer smoking daily. Russia shows a balanced prevalence for those families with higher affluence. Russian boys tend to have a high prevalence of smoking weekly in high affluence families as compared to girls.

**Figure 4:**
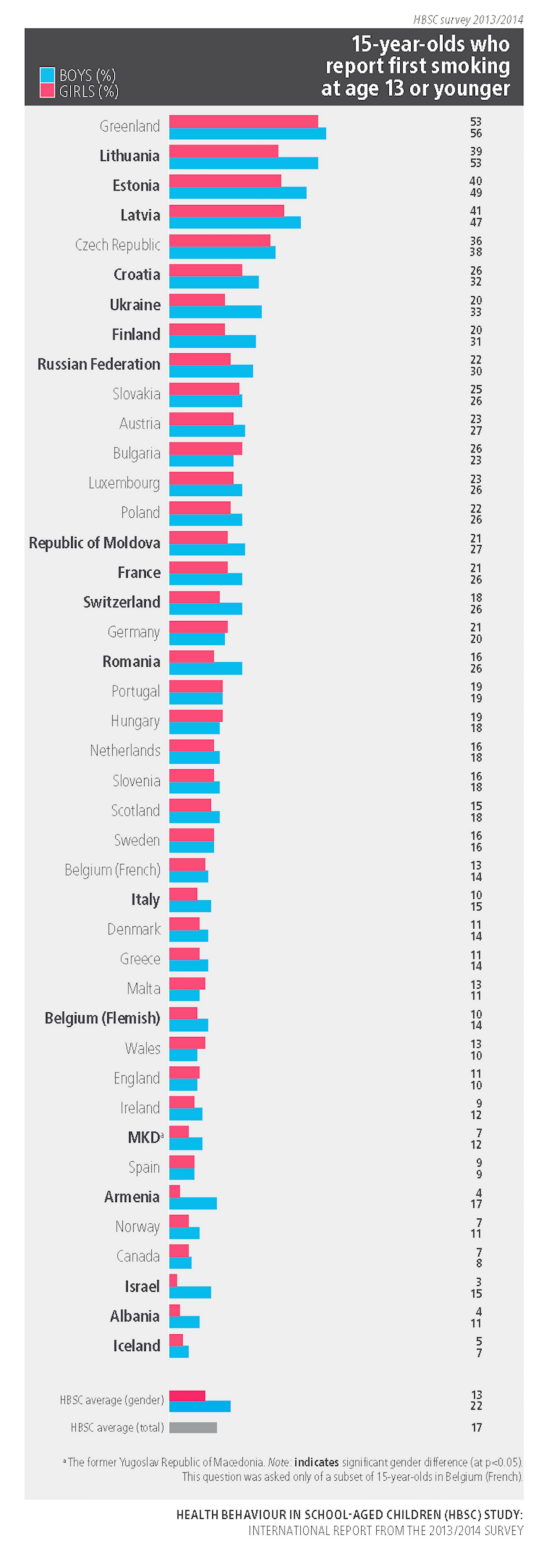
The proportion of young people who reported smoking cigarettes at the age of 13 or younger for 15 years old Copyright © World Health Organization (WHO) Regional Office for Europe, 2016. All rights reserved. Images and other multimedia content used with permission.

Figure 4 represents the proportion of young people who reported smoking cigarettes at the age of 13 or younger for 15-year-olds. In Russia, boys recorded 30% for those who smoked at age of 13 and below as compared to girls who were at only 22%. In almost all countries, a significant gender difference was observed, with smoking more prevalent in boys.

**Figure 5:**
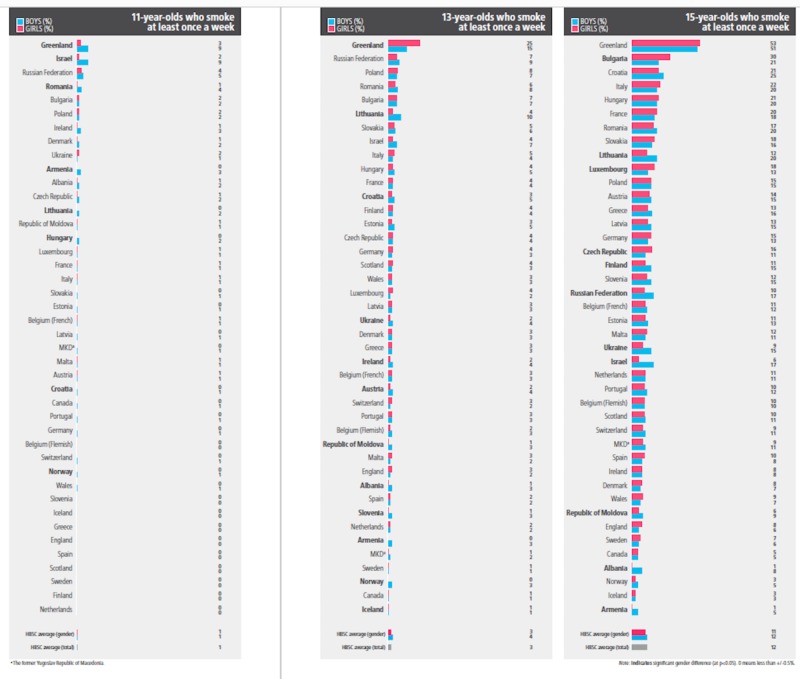
11, 13, and 15-year-olds who smoke at least once a week Copyright © World Health Organization (WHO) Regional Office for Europe, 2016. All rights reserved. Images and other multimedia content used with permission.

Figure 5 shows the bar chart for 11, 13, and 15-year-olds who smoke at least once a week. In Russia, the prevalence of regular weekly smoking increased from the 11-year-olds to the 15-year-olds. Similar results were observed in all the countries except for boys in Armenia and some girls in Albania, Armenia, and Norway.

Health consequences

Tobacco use remains one of the deadliest habits, with smoking being known to kill half of the entire generation of tobacco users [8]. According to Drope J, Schluger N, Cahn Z, et al., over 310,000 Russians pass away every year from smoking-related chronic disorders. Approximately a quarter of all male and 6% of female deaths in the Russian Federation are related to smoking. This accounts for 15% of all deaths in Russia. Nearly 80% of tracheal, bronchus, and lung cancer mortalities are smoking related. Again, 61% of the chronic obstructive pulmonary disease deaths and 19% of ischemic heart syndrome originate from tobacco use [8].

It was shown that active and passive smoking in adults and children is one of the main risk factors for cardiovascular diseases and the development of atherosclerosis and has a significant effect on the increase in pulse wave velocity (PWV) [22].

PWV is of the highest significance for assessing arterial remodeling using non-invasive diagnostics methods. The values of PWV were determined for the first time in the population of Russian children by non-invasive oscillometric arteriography using the “TensioMed” device (TensioMed Kft, Hungary). It was determined that in 36 Russian children (11%), both boys and girls, mostly 9-11-year-olds, the level of PWV was higher than the 9 percentile of the international normal value, which is a reflection of the increased risk of cardiovascular diseases in children of this age in the Russian population and may ultimately determine the catastrophically high mortality rate of Russians from cardiovascular diseases [1]. A possible explanation for this window may be the early age of the onset of active and passive smoking of tobacco among Russian schoolchildren (nine years old) [23].

Bowatte et al. (2015) in their research suggest that active smoking was significantly related to asthma. The studies further revealed that exposure to second-hand forms of smoking among most of these children is strongly associated with asthma attacks [24]. Clinical studies and observation studies further revealed the common effects of these forms of smoking, including throat irritation, chest pain, and coughing among patients [25]. The studies continued to confirm that these symptoms may be severe among those asthmatic patients who are exposed to smoking. The health consequences are really worrying to society.

Costs to society

About 15% of the aggregate health care expenditure in high-income countries can be attributed to smoking. A society dominated by tobacco use is exacted with high cost implications. According to Lunze & Migliorini, the amount of money used on tobacco products in 2009 was 1% of the country’s gross domestic product (GDP). Tobacco use lowers productivity as a result of the early deaths caused by tobacco-related sickness. Tobacco use has been shown to cost the nation at least RUB 710.4 billion, which is equivalent to 23.7 billion US Dollars. This amounts to more than 3% of the Russian Government’s GDP. Tobacco users incur significant costs to treat smoking-related sickness. Cardiovascular and respiratory diseases are attributed to smoking, which alone costs the Russian healthcare system nearly RUB 125 billion, equivalent to 4.2 billion U.S Dollars [26].

Current tobacco control and smoking prevention policy in the Russian Federation

A challenging opportunity for the effective and efficient reduction of the country's tobacco problem is contained in the Russian "National Tobacco Control Concept" and the latest Ministry of Health and Social Development (MoHSD) project for a novel tobacco control bill. Recent reports show that the resilient tobacco industry impacts efforts to reduce impending tobacco control actions.

Russia should work on strengthening the national policy that controls tobacco by setting up vibrant, evidence-based health communications to implement the obligations of the Framework Convention on Tobacco Control and "National Tobacco Control Concept" and implement its hopeful bill. Even though Russia lacks home-based, unprocessed tobacco manufacturers whose source of revenue will be disadvantaged by the policy, the move represents the global consideration and interests from international tobacco firms. The significant policy expects opposition, largely from cigarette manufacturers and the allied export and supply companies. However, leaders need to relate to the current "National Tobacco Control Concept” to look for constant political commitments to form robust alliances for advancing control of tobacco consumption within the nation, specific attention being on tobacco use among youths and women.

According to Andreeva et al., smoke-free policies remain an effective tool for protecting non-smokers from indirect smoke contact, as well as an opportunity for smokers to abandon tobacco. Previous studies revealed a decline in the frequency of present tobacco use among universities and schools running smoking-free. The research discovered that smoking-free institutions assist young people in evading normal smoking behaviors. This is achieved by facilitating a willingness to leave smoking and intensify the attempts to quit, which in turn leads to a large proportion of current cigar smokers and those intending to start to turn into non-smokers [27].

The fight against smoking in Russia has just started and the government has come up with several ways to reduce the number of people smoking. Current Russian state laws prohibit smoking and the use of electronic smoking devices in all indoor public places. Consequently, the government has come up with policies that restrict smoking in schools, as the government thinks that students may be influenced by seeing their peers smoking [26]. This law also prohibits smoking within 100 meters of entrances, exits, windows that open, and ventilation intakes that serve enclosed areas where smoking is prohibited.

Bringing together different stakeholders and coordinating their partnership has the likelihood of gaining force past the existing policy opposition and the influence of interested industries. A positive tobacco control policy change, together with successful changes, can determine the capacity of the Russian government for improving public health and managing the current health challenges.

Notably, the policy taken by the Russian government has helped to reduce the number of smokers among 14-16-year-old teenagers. The classes of maternal smoking cessation that were introduced in Moscow are well-known to be the most effective of the measures that are community-based, as they lead to a quit rate of up to 35% despite there being high costs incurred as compared to other measures like self-help quit-smoking kits. We hope that “community-oriented” approaches will help protect society against the negative vices that may be created by cigarettes. Further, this approach is believed to help in reducing progressive smoking among adolescents and young adults.

Role of heated tobacco products in asthma development and exacerbation

As stated by Ng et al., smoking prevalence has decreased but the number of cigarette smokers worldwide has increased due to population growth [28].

Since 1980, large reductions in the estimated prevalence of daily smoking were observed at the global level for both men and women, but because of population growth, the number of smokers increased significantly.

Heated tobacco products (HTPs) are now the most commonly used tobacco products among youth in Moscow, suppressing conventional cigarettes in 2015-2016. Unfortunately, there exists a huge gap when it comes to knowledge about the HTPs since the products have not been on the market for long. Currently, HTPs products are considered to be as harmful as conventional cigarettes.

Additionally, limited knowledge is available with regards to using the products to help smokers to quit or even products that have the potential to trigger asthma attacks. Nevertheless, no evidence links the reduction in exposure to a reduction in asthma exacerbation [29]. Consequently, there have been calls for independent research to be done to bring clarity to the issue. When it comes to second-hand exposure, inadequate evidence exists on the potentially detrimental effects of using HTPs among patients with BA. The WHO recommends that HTPs need to be subjected to the policy and regulatory procedures applicable to similar tobacco goods, as stipulated in the WHO Framework Convention on Tobacco Control [30].

Correlations in research results

In accordance with the above-mentioned facts, we have discussed the relationship between smoking and asthma among adolescents, to comprehend the risks involved. From the data and the results of our study, 25.3% of the respondents said that they have ever smoked for as long as a year. Among the participants who had ever smoked, most of them started smoking at the age of 16 years while the state-reported average age for a new smoker is 11.4.

The lower rates of smoking among study participants could also reflect the success of prevention efforts targeting school-age children. We believe that recent policy changes (i.e., the enforcement of minors' access laws and legislation restricting smoking in public places, schools, and universities) and the dissemination of relevant data to the public contributed to the decline in social acceptance of smoking among teenagers and young adults.

The data analyzed do not provide sufficient evidence to show that there is a causative association between smoking status and the development of asthma, hay fever, or sinusitis. Therefore, despite the popular belief that smoking causes conditions like allergic rhinitis, allergic dermatitis, and food allergy and asthma, the data analyzed do not support that. The somewhat conflicting results reported in the literature on the impact of smoking on asthma and our data may be explained by the fact that patients recruited in clinics generally have a more severe form of asthma and, therefore, do not represent the several phenotypes of the disease that can be found in the general population. In order to generalize the findings, there is a need to increase statistical power through an increased sample size.

If an outcome is rare or if the results are inconclusive, it is difficult for a single study – even a relatively large-scale one – to detect that difference, making it unlikely that the results will reach statistical significance. In this case, a meta-analysis is supposed to be used to increase the sample size, thereby increasing the power of the analysis.

In addition to the young age of our study population and the relatively low exposure to active smoking, the healthy smoker effect could explain the lack of significant association between smoking status and the prevalence of asthma.

From the analysis that has been done, it is evident that there is a clear link between second-hand smoke and asthma in children. From the findings, there is no association between a father smoking regularly during childhood and the child being hospitalized before the age of two years for lung disease. However, there was an association between mothers regularly smoking during childhood or before birth and the child being hospitalized before the age of two years for lung disease. The parents of the participants in this study were married, heterosexual men with biological children, who worked outside the home, and the mother stayed home as primary caretaker and did not contribute financially. As far as we understood, stay-at-home mothers were rarely away from their children.

Also, children whose mothers smoke regularly during their childhood or before their birth are at a higher risk of being hospitalized before the age of two years for lung disease as compared to those children whose mothers did not smoke during the child’s childhood or before birth. Parents smoking during early adolescence and mothers smoking during pregnancy may independently increase asthma risk in offspring. Thus, risk factors for asthma should be sought in both parents and before conception.

Unfortunately, smoking cessation programs by the government that entail educating and offering pharmaceutical therapies to the maternal smokers are not well -founded. For non-compliant pregnant women in Russia, there is no significant public pressure and anti-smoking community prevention programs: pregnant women are left to make their own decisions based on their values, circumstances, and preferences. Maternal smoking during pregnancy has a large contribution to childhood asthma among the large population that resides in the Moscow area and all over Russia. This poses a risk to society, as there are high chances of having a society with its largest population ailing from asthma and other chronic diseases that result from maternal smoking during pregnancy.

Further efforts should be devoted to encouraging smoking cessation in patients with asthma who continue to smoke and to develop strategies for preventing tobacco use in young patients. Looking at the rates, which are also proportions, there is a need for more focused smoking-control measures. There are stark disparities among different groups’ smoking prevalence rates. Tobacco control efforts should be targeted at specific groups that have a higher proportion of their population who are engaging in smoking. These groups include people with low levels of education and those people who are living far below the federal poverty level.

## Conclusions

In summary, we have analyzed the data concerning smoking and its effects and conveyed the facts argued to gain a comprehensive understanding of smoking and its impact. Our research indicates a moderate decrease in smoking prevalence among Russian teenagers. The fact that the smoking rates among study participants are relatively low is quite encouraging, and anti-smoking campaigns should be intensified to drive the rates lower. Since there is no clear association between smoking status and the development of any atopic disorders, it is imperative to say that smoking cessation will have very little effect on allergy prevalence. According to our findings, maternal smoking remains an outstanding issue among pregnant women in Russia, and children whose mothers smoked are especially vulnerable to asthma and other respiratory disorders. This highlights the importance of establishing effective and well-structured programs for smoking cessation in Russia.
